# Leiomyosarcoma of the Duodenum: A Case Report on a Rarely Encountered Tumor

**DOI:** 10.7759/cureus.65182

**Published:** 2024-07-23

**Authors:** Sakshi S Dudhe, Gaurav V Mishra, Pratapsingh Parihar, Devyansh Nimodia, Anjali Kumari, Dhananjay Shinde

**Affiliations:** 1 Radiodiagnosis, Datta Meghe Institute of Higher Education and Research, Wardha, IND

**Keywords:** leiomyosarcoma, neoplasm, gastrointestinal, rare, duodenum

## Abstract

Leiomyosarcoma (LMS) is an extremely rare malignant pathology affecting smooth muscle cells, with the uterus being the predominant location of LMS. Its occurrence in the duodenum is rare, making it a diagnostic challenge for radiologists. Patients with duodenal LMS can present with very vague symptoms such as abdominal discomfort, loss of weight, or manifestations associated with internal gastrointestinal bleeding. In this case report, we have an 82-year-old female presenting with duodenal LMS, which is a very atypical location. An esophagogastroduodenoscopy and further workup revealed a duodenal mass, which was biopsied. The lump was identified as an LMS using immunohistochemistry and histopathology. Despite its rarity, it presents diagnostic and therapeutic challenges due to its nonspecific clinical manifestations and radiological findings. By exploring the existing literature and clinical insights, we aim to provide a comprehensive understanding of this rare condition, highlighting the need for interdisciplinary collaboration and tailored therapeutic strategies to diagnose and manage this disease entity effectively.

## Introduction

Leiomyosarcomas (LMSs) are infrequent malignant neoplasms originating from the mesenchyme. They can develop at any location in the gastrointestinal tract, with a higher prevalence in the stomach and a lower prevalence in the duodenum. Patients with LMS tend to present between 50 and 70 years of age, with females being more affected than males [1]. LMSs tend to be larger than the leiomyomas and more frequently involve the second part of the duodenum [2]. Duodenal LMSs primarily occur in the second part, with the remaining cases distributed among the third, first, and fourth parts in descending order of prevalence. These tumors commonly demonstrate a tendency to increase in size in an outward direction from the lumen [3]. The most common symptom, in general, was abdominal pain. Numerous characteristics were noted for this pain, including ulcer-like, cramping, persistent, monotonous, dull, and vague, with or without gastrointestinal bleeding. Addressing the pathophysiology of LMS, it is unclear from the instances evaluated whether the tumors originated de novo or as a result of alterations to the growth of preexisting leiomyomas [4]. The existing standards used in identifying primary LMS include exhibiting positive reactivity to smooth muscle antigens, lack of response to gastrointestinal stromal tumor immunomarkers (CD117, CD34), and specific histomorphologic features [5]. The diagnosis of duodenal LMS usually entails a combination of imaging investigations, histological examination, and clinical evaluation. The cornerstone of treatment is surgical removal, which aims to remove the tumor completely with negative margins. Depending on the size and location of the tumor, the extent of surgery may vary, ranging from local excision to pancreaticoduodenectomy (Whipple technique) for periampullary tumors.

## Case presentation

An 82-year-old female presented to the out patient department with six months history of dull boring upper abdominal pain radiating to lower abdominal quadrants. Other symptoms she experienced included gastroesophageal reflux, diarrhea, weight loss, mild breathlessness, painful micturition, and generalized weakness. Despite over-the-counter antacids for her gastrointestinal reflux, symptoms persisted, prompting her to seek medical attention. She had no history of drinking or smoking. The laboratory tests, such as liver function tests and total blood counts, were advised and were unremarkable. A stool occult blood test was negative, which ruled out gastrointestinal bleeding. Esophagogastroduodenoscopy was done, and it revealed mild luminal constriction by mass, mainly in the second part of the duodenum. A number of biopsies were taken throughout the process. The biopsy specimens underwent histopathological analysis and revealed spindle-shaped cells with prominent nuclear atypia and enhanced mitotic activity, which is compatible with an LMS diagnosis. The origin of the tumor cells as smooth muscle was verified by immunohistochemistry. The diagnosis of LMS was confirmed by the spindle cells showing positive expression for smooth muscle actin and desmin but negative expression of CD117, DOG1, CD34, and S 100. Alpha-fetoprotein, carcinoembryonic antigen, and cancer antigen-125 were among the tumor markers that were found to be within normal ranges.

**Figure 1:**
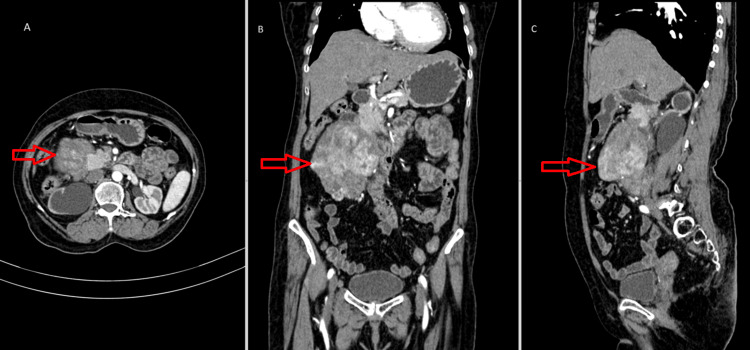
CECT of the abdomen of arterial phase in axial (A), coronal (B), and sagittal (C) sections, with red arrows showing large heterogeneously enhancing exophytic mass lesions along the second and third parts of the duodenum, measuring approximately 10 x 9.7 x 6.6 cm, which was proven histopathologically to be LMS of the duodenum CECT: contrast-enhanced computed tomography; LMS: leiomyosarcoma

To assess the extent of the disease, the patient underwent contrast-enhanced computed tomography (CT) scans of the abdomen and CT urography. These revealed a heterogeneously enhancing exophytic mass lesion arising from the second and third parts of the duodenum measuring approximately 10 x 9.7 x 6.6 cm (Figure 1).

**Figure 2:**
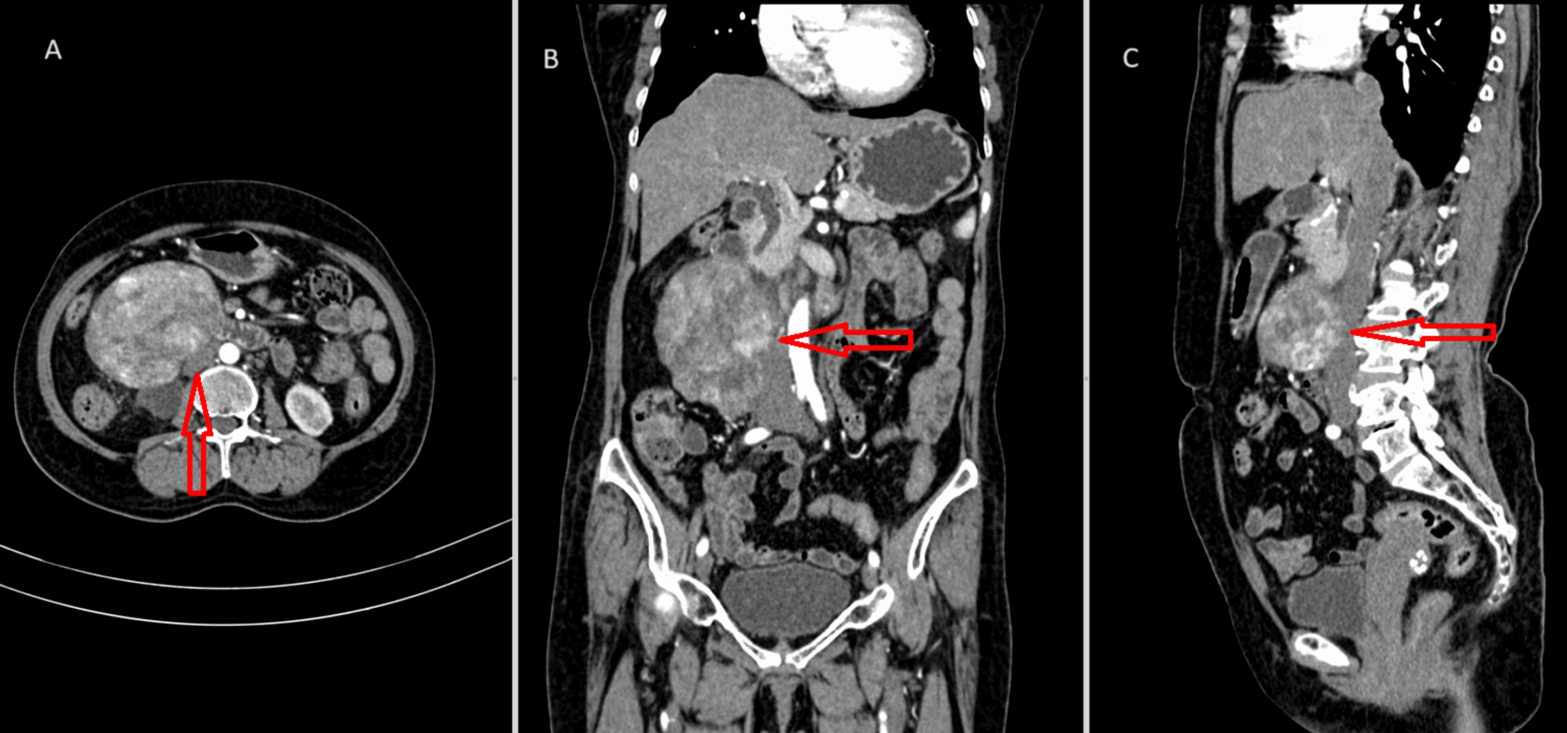
CECT of the abdomen in axial (A), coronal (B), and sagittal (C) sections, with red arrows showing lesions causing mass effect on IVC CECT: contrast-enhanced computed tomography; IVC: inferior vena cava

**Figure 3:**
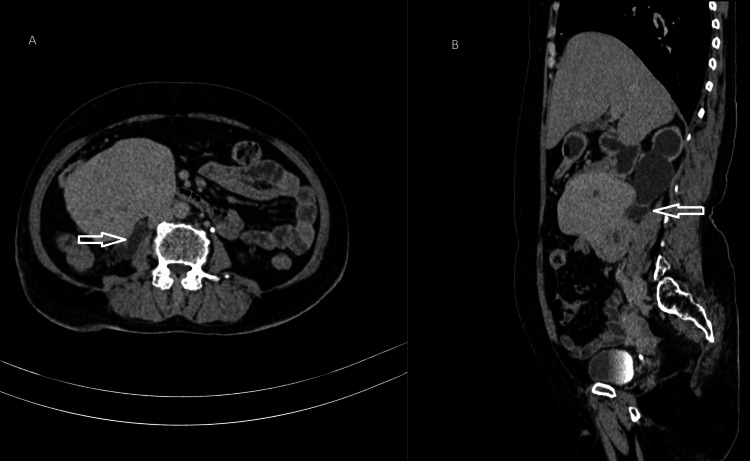
CT urography images of the axial (A) and sagittal (B) sections in the excretory phase showing mass lesions compressing the right proximal ureter (white arrows), causing upstream hydroureteronephrosis. Also, there was no contrast excretion into the right ureter, suggesting a nonfunctioning right kidney, whereas the left ureter showed good contrast excretion CT: computed tomography

Posteriorly, the mass depicts a loss of fat planes with an inferior vena cava (Figure 2) and a compressing right upper ureter (Figure 3).

**Figure 4:**
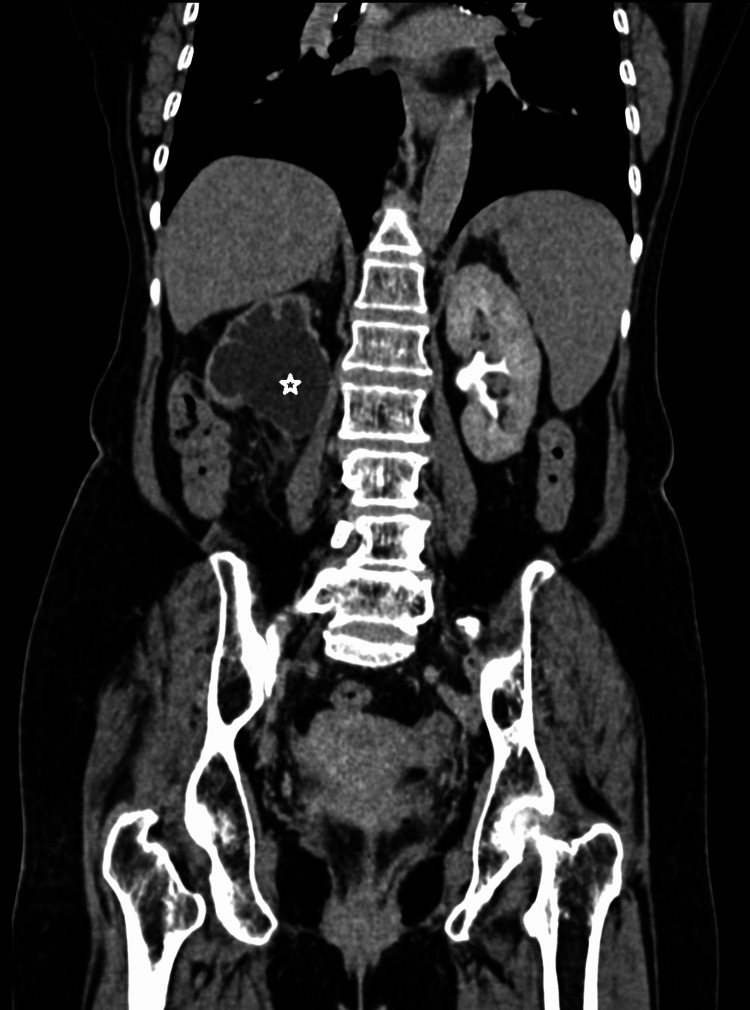
CT urography image in a coronal section of the excretory phase, showing no contrast excretion in the right kidney with upstream grade 4 hydroureteronephrosis (star) due to compression by mass lesion. The left kidney shows normal contrast excretion CT: computed tomography

There was resultant gross dilatation of the right upper ureter and pelvicalyceal system suggestive of upstream right gross hydroureteronephrosis. Since there was pelvicalyceal dilatation, total loss of medulla, overlying cortical thinning, and dilatation of the right upper ureter, a diagnosis of grade 4 hydroureteronephrosis was made (Figure 4).

**Figure 5:**
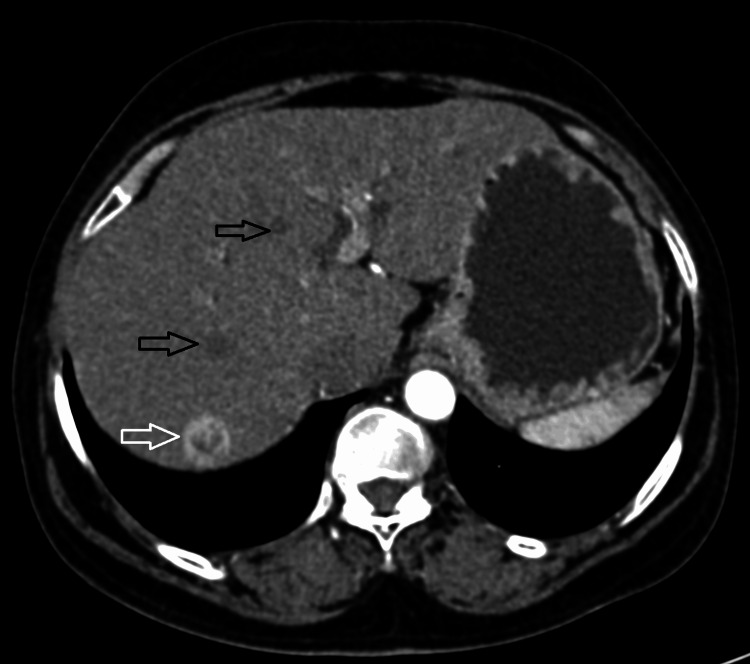
CECT image of arterial phase in axial section, showing hypervascular lesion in segment VII of the liver, likely hypervascular liver metastasis (white arrow). Few hepatic cysts were also noted (black arrows) CECT: contrast-enhanced computed tomography

The right kidney was declared nonfunctioning due to no uptake of contrast on corticomedullary, nephrographic, and 15-minute delayed excretory phases of CT urography. The common bile duct was prominent, likely secondary to compression by the mass lesion at the ampulla. Hypervascular lesions were noted in the liver suggestive of hypervascular metastasis from the primary mass lesion, i.e., histopathologically proven LMS. Few hepatic cysts were also noted in the liver (Figure 5).

An incidental finding of calcified subserosal fibroid measuring 1.7 x 1.2 cm was also noted in the uterus. After a multidisciplinary discussion involving gastroenterologists, surgeons, and oncologists, the decision was made to proceed with surgical resection of the duodenal tumor. The patient underwent a pancreaticoduodenectomy (Whipple procedure) to achieve complete removal of the tumor with negative margins. The pathology analysis of the surgically removed duodenal mass confirmed the presence of LMS of duodenal origin. Regular follow-up visits and chemoradiotherapy were scheduled for the patient to monitor for any signs of disease recurrence or progression. Periodic imaging studies, including CT scans, were planned to assess for local recurrence or any other distant metastasis.

## Discussion

Duodenal LMS is an infrequently encountered tumd the very first case of duoor, with only a limited number of cases reported in the literature. Its clinical presentation can mimic various gastrointestinal disorders, making it a diagnostic dilemma. Gastrointestinal tract LMS is associated with bleeding, pain, mass obstruction, altered bowel habits, jaundice, fever, and chills [6]. Duodenal LMSs are seen in the 50-70 age group with a slight female dominance of this tumor. The very first case of duodenal LMS was reported in 1920. Until today, only close to 200 cases have been observed in the literature. In only 40% of cases, an abdominal mass is palpable. The symptoms observed are often attributed to the tumor's tendency to grow externally. Additionally, these tumors exhibit elevated vascularity. In 40% of cases, there is a palpable abdominal lump. The tumor's propensity to develop extraneously was identified as the cause of the symptoms. There is also a high vascularity in these tumors. LMSs are often diagnosed at advanced stages of the disease due to the indolent course of the illness [7]. Symptoms of obstruction, gastrointestinal bleeding, or abdominal pain are typical symptoms of duodenal tumors. Large size, limited resections, and local recurrence were associated with poor outcomes [8]. Similar to other soft-tissue sarcomas, LMSs can spread hematogenously, which can include the liver and lungs; lymphatic dissemination is less common. Diagnosing duodenal LMS is difficult because it is an uncommon tumor [1]. Reviews of the literature previously highlighted the propensity of LMSs to experience ulceration and necrosis, as well as to appear as an unexplained source of gastrointestinal hemorrhage [9]. Macroscopically, the excised portion shows a gentle grayish-yellow hue with a distinctive whorled pattern, frequently depicting central necrosis and hemorrhage. The neoplasm has a tendency to surpass its vascular supply, leading to manifestations such as ulceration, degeneration, necrosis, hemorrhage, cavitation, sinus and fistula formation, as well as potential infection. Occasionally, evidence of calcification is observed. Under microscopic examination, a discernible capsule is commonly observed, albeit morphologically variable, while the predominant cellular constituents are spindle-shaped with hyperchromatic nuclei typically arranged in palisades. Detection of myofibrils can be achieved through staining with phosphotungstic acid-hematoxylin. Mitotic activity is frequently noted in varying quantities, yet it is widely agreed that the presence of mitotic figures does not correlate with the malignant potential of the tumor. LMS of the duodenum primarily disseminates through local infiltration; however, hematogenous spread may lead to metastasis in the liver, peritoneum, and omentum. Lymphatic dissemination is considered rare [10].

Clinical latency is the reason for which the diagnosis is often done at an advanced stage. Surgery should be performed aggressively to remove the tumor and its surrounding extensions [11]. The predominant radiological abnormality observed in tumors is a filling defect located centrally, often associated with ulceration or fistula formation. This is further supported by a common gross pathologic feature of central necrosis in the tumor, which plays a significant role in explaining various clinical manifestations and the radiographic appearance of the neoplasm. Metastasis in duodenal LMS primarily occurs through local invasion. Radical local excision of the tumor has proven to be the most successful surgical intervention. Extensive removal of regional lymph nodes is not typically recommended, given the infrequent occurrence of nodal metastases. Advances in pancreatic and duodenal surgical techniques have enabled excisional therapy with a low mortality rate [4]. The prognosis is unfavorable, with a mean survival of 50 months and a 50% five-year survival rate. After resection, the 10-year survival rate is about 50%, while it is just 10% in the absence of resection [1].

## Conclusions

This case study of duodenal LMS emphasizes the significance of considering uncommon malignancies in the differential diagnosis of gastrointestinal symptoms. The patient's manifestation with recurrent abdominal pain and gastrointestinal reflux brings attention to the nonspecific nature of symptoms linked with duodenal LMS, often resulting in diagnostic complexities and delays. Despite the obstacles in diagnosis, timely implementation of a pancreaticoduodenectomy enabled complete tumor removal, demonstrating the pivotal role of surgical intervention in achieving positive outcomes for patients with this rare malignancy. Nonetheless, the cautious prognosis and likelihood of disease recurrence underscore the importance of diligent long-term monitoring. Furthermore, this case highlights the requirement for a collaborative approach involving gastroenterologists, surgeons, oncologists, and pathologists in the assessment and treatment of duodenal LMS. Cooperation among these specialized fields is essential for precise diagnosis, optimal treatment strategies, and holistic patient management. In summary, this case study adds to the expanding body of research on duodenal LMS and stresses the significance of early identification, timely intervention, and interdisciplinary teamwork in enhancing patient care for this rare malignancy.
